# Discovery of *Novaculina myanmarensis* sp. nov. (Bivalvia: Pharidae: Pharellinae) closes the freshwater razor clams range disjunction in Southeast Asia

**DOI:** 10.1038/s41598-018-34491-8

**Published:** 2018-11-05

**Authors:** Ivan N. Bolotov, Ilya V. Vikhrev, Manuel Lopes-Lima, Zau Lunn, Nyein Chan, Than Win, Olga V. Aksenova, Mikhail Yu. Gofarov, Alexander V. Kondakov, Ekaterina S. Konopleva, Sakboworn Tumpeesuwan

**Affiliations:** 1 Northern Arctic Federal University, Arkhangelsk, Russia; 2 Federal Center for Integrated Arctic Research, Russian Academy of Sciences, Arkhangelsk, Russia; 3 CIIMAR - Interdisciplinary Centre of Marine and Environmental Research, University of Porto, Matosinhos, Portugal; 4 CIBIO/InBIO - Research Center in Biodiversity and Genetic Resources, University of Porto, Vairão, Portugal; 5 Fauna & Flora International – Myanmar Program, Yangon, Myanmar; 6 Department of Zoology, Hpa-An University, Hpa-An, Kayin State Myanmar; 7 Department of Biology, Faculty of Science, Mahasarakham University, Maha Sarakham, Thailand

## Abstract

The razor clam genus *Novaculina* represents an example of a marine-derived, secondary freshwater group. It was thought to comprise three species: *N. gangetica* (Ganges and smaller basins in Bangladesh and northwestern Myanmar), *N. siamensis* (Bang Pakong and Pasak rivers in Thailand and Mekong River in Vietnam), and *N. chinensis* (lower Yangtze River, China). Here we describe *Novaculina myanmarensis* sp. nov., an additional species from the Ayeyarwady and Salween basins representing a divergent lineage that appears to be sister to *N. gangetica*. This new record closes a *Novaculina* range disjunction between northwestern Myanmar and Thailand. The populations of this novel species share a shallow molecular divergence from each other indicating potential dispersal events between the two distant freshwater basins during the Late Pleistocene. Our ancestral area modeling suggests that the MRCA of *Novaculina* crown group was a salt-tolerant freshwater species. The recent *Novaculina* species most likely originated via allopatric speciation. Our findings highlight that generalist estuarine species could have played the role as a source for bivalve expansions into freshwater and that western Indochina is a separate biogeographic subregion, which is clearly distinct from India. A new synonymy is proposed as follows: Pharellinae Stoliczka, 1870 = Novaculininae Ghosh, 1920 syn. nov.

## Introduction

Freshwater bivalves are a taxonomically diverse ecological group, which includes representatives of at least 19 families^1,2^. Unionida is the only strictly freshwater order among Bivalvia representing a monophyletic entity with six families, i.e. Unionidae, Margaritiferidae, Etheriidae, Iridinidae, and Mulleriidae^2,3^. However, several other orders have small to large radiations in freshwater, e.g. Venerida, which includes families such as Cyrenidae, Dreissenidae, Sphaeriidae, and Pharidae^1^.

Pharidae is a primary marine family^4^, but it contains a single typically freshwater genus, *Novaculina* that was thought to include three species: *N. gangetica*, *N. siamensis*, and *N. chinensis*. This genus belongs to the subfamily Novaculininae, which also comprises a second genus with two species, *Sinonovacula constricta*^5^ and *S. mollis*. Annandale^6^ suggested that *Novaculina* is a relict marine-derived freshwater lineage, and this hypothesis has recently been supported by multi-locus phylogenetic analyses^4^.

*Novaculina gangetica* was considered an endemic species of the Ganges River system in India and Bangladesh^7,8^, but it was recently discovered in the Kaladan and Lemro rivers in northwestern Myanmar^4^. *Novaculina siamensis* was known from the Bang Pakong and Pasak rivers in Thailand^9,10^, but Sayenko *et al*.^11^ found this species in the Mekong Delta in Vietnam. Finally, *Novaculina chinensis* was described from the Taihu Lake, a large floodplain water body in the lower Yangtze River basin^12^, and later reported from two additional lakes and a river in the same region^13–15^.

**Figure 1:**
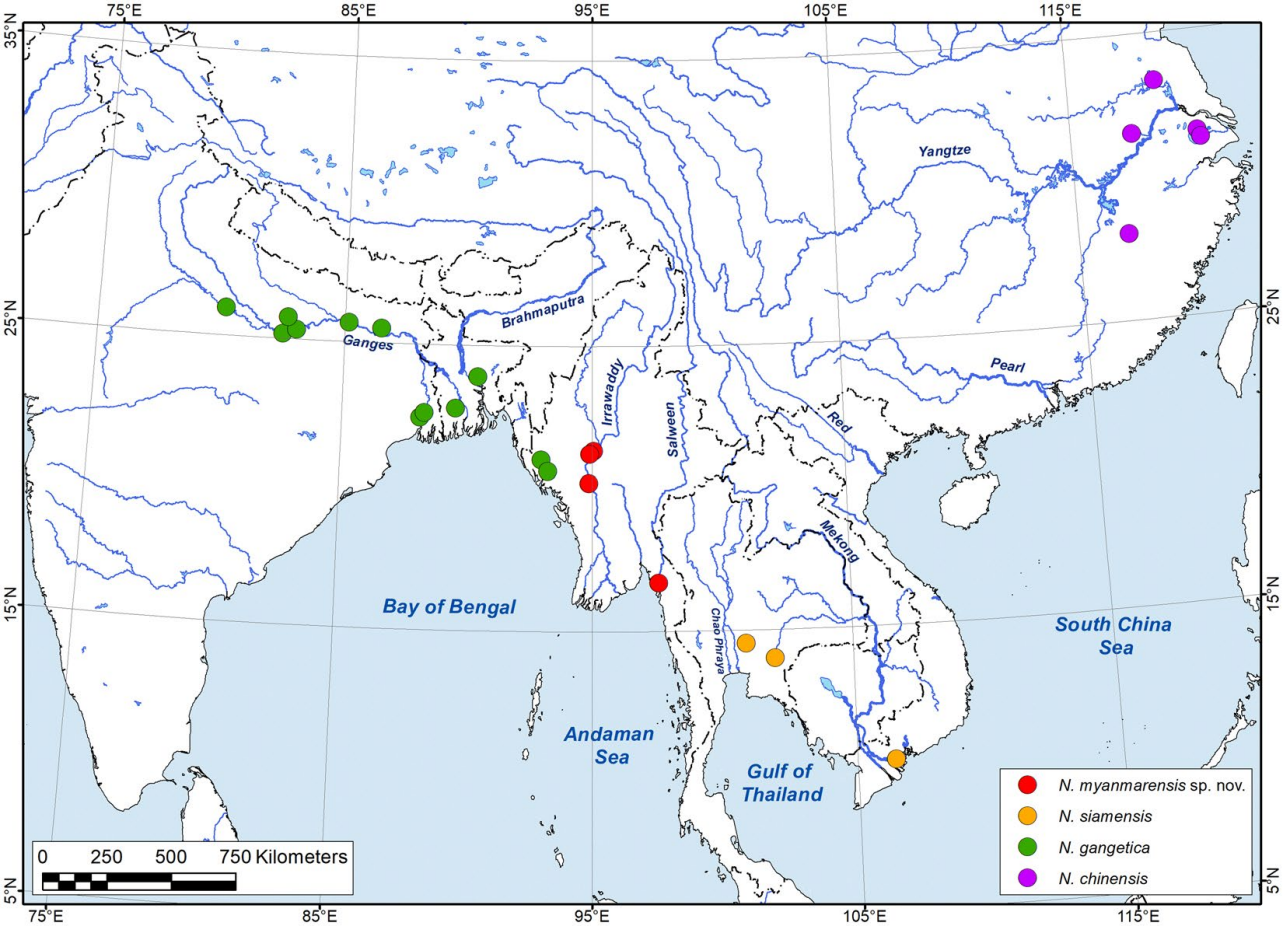
Distribution range of the genus *Novaculina* Benson, 1830 based on available georeferenced records (Supplementary Table 2). The map was created using ESRI ArcGIS 10 software (www.esri.com/arcgis); the topographic base of the map was created with Natural Earth Free Vector and Raster Map Data (www.naturalearthdata.com) and Global Self-consistent Hierarchical High-resolution Geography, GSHHG (http://www.soest.hawaii.edu/wessel/gshhg/). (Map: Mikhail Yu. Gofarov).

A large *Novaculina* range disjunction was situated in central and eastern Myanmar (Ayeyarwady and Salween river basins) (Fig. 1). However, we have discovered an additional species in this genus during a recent field trip to Myanmar. The present study aims to describe a new species, *Novaculina myanmarensis* sp. nov., to provide a brief taxonomic overview of all *Novaculina* species, and to discuss the putative origin of freshwater lineages in estuarine bivalves within a broad phylogenetic and biogeographic context.

## Results

### Multi-locus phylogeny of the Pharidae

**Figure 2:**
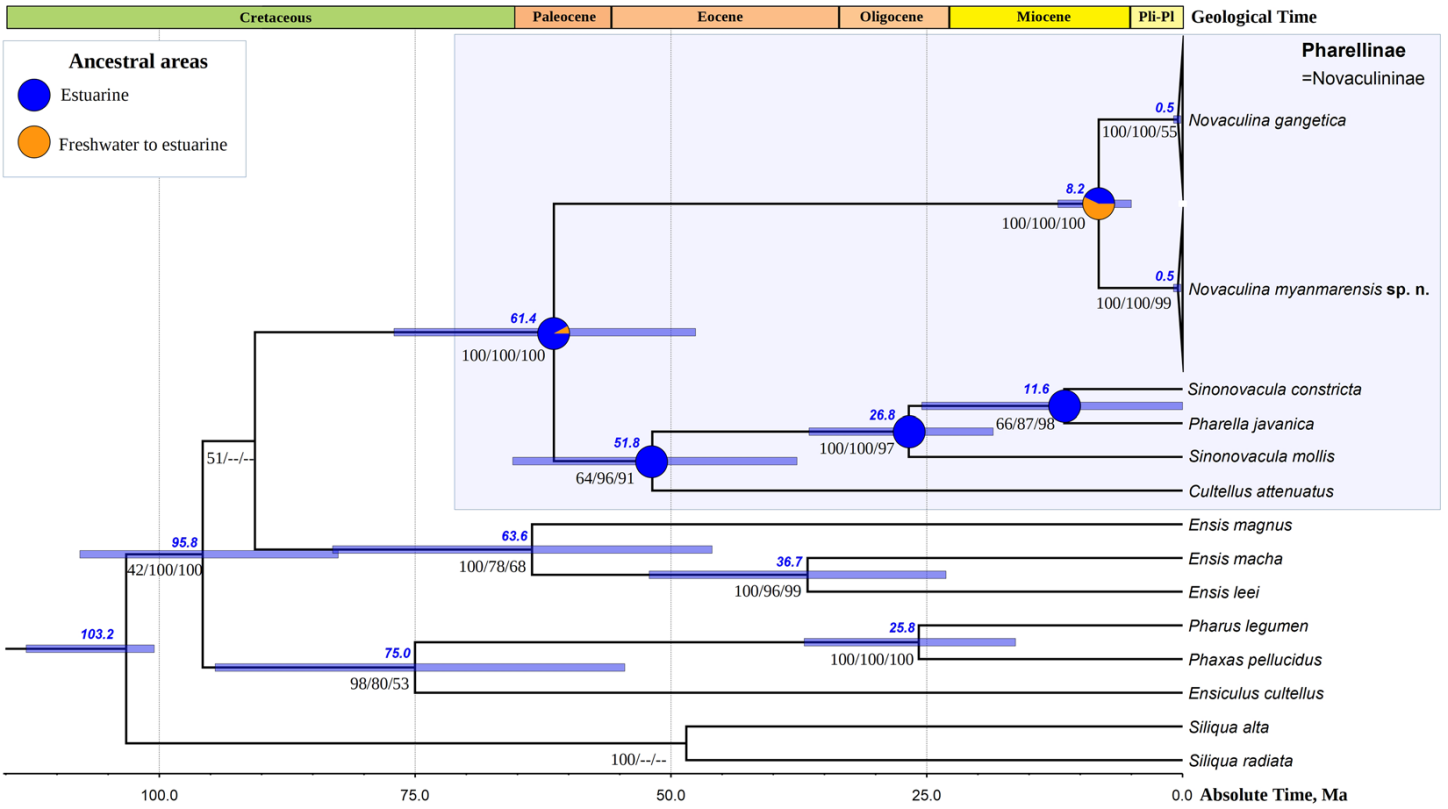
Fossil-calibrated chronogram of the Pharidae computed under a lognormal relaxed clock model and a Yule process speciation implemented in BEAST 1.8.4 and obtained for the complete data set of mitochondrial and nuclear sequences (five partitions: three codons of COI + 16S rRNA + 28S rRNA). Bars indicate 95% confidence intervals of the estimated divergence times between lineages (Ma). Black numbers near nodes are BPP values of BEAST model/BPP values of MrBayes model/BS of RAxML model. Blue numbers near nodes are mean ages (Ma). Stratigraphic chart according to the International Commission on Stratigraphy, 2018. The node pies indicate ancestral area reconstructions (probability of each area combination) in accordance with the combined biogeographic model (combination of the S-DIVA + DEC + S-DEC models). Age values for weakly supported nodes are not shown.

Our multi-locus phylogeny (five partitions: three codons of COI, 16S rRNA, and 28S rRNA) indicates that *Novaculina myanmarensis* sp. nov. and *N. gangetica* represent phylogenetically distant lineages belonging to a separate, fully-supported subclade (Fig. 2). The mean *p*-distances (±standard error estimates) between the new species and *Novaculina gangetica* are as follows: COI = 8.0 ± 1.0%, and 16S rRNA = 1.9 ± 0.6%. There is a single nucleotide substitution in the nuclear 28S rRNA gene between these species. The *Novaculina* subclade is fully supported by both Bayesian and maximum likelihood models, and it appears to be closely related to another Pharidae subclade, which includes representatives of *Sinonovacula*, *Pharella javanica*, and *Cultellus attenuatus*. *Pharella javanica* belongs to the *Sinonovacula* subclade, and this pattern is strongly supported by our models, indicating the synonymy of Pharellinae Stoliczka, 1870 and Novaculininae Ghosh, 1920.

### Divergence times

Our fossil-calibrated phylogeny suggests that the crown group of the Pharidae has been originated in the mid-Cretaceous (mean age = 103 Ma, 95% HPD 100–113 Ma) (Fig. 2). The Pharellinae (=Novaculininae) clade most likely originated in the Paleocene (mean age = 61 Ma, 95% HPD 48–77 Ma). The origin of the *Novaculina* crown group placed in the Miocene (mean age = 8 Ma, 95% HPD 5–12 Ma). Finally, the crown group of *Pharella* + *Sinonovacula* clade most likely originated in the Oligocene (mean age = 27 Ma, 95% HPD 19–36 Ma).

### Ancestral areas

The ancestral area modeling indicates that the most recent common ancestor (MRCA) of the Pharellinae (=Novaculininae) clade was an estuarine species (probability 92.1% by integrative model, 100% by S-DIVA model, 87.5% by DEC model, and 88.8% by S-DEC model). The MRCA of the *Novaculina* crown group was most likely a salt-tolerant freshwater species like its recent descendants (probability 56.9% by integrative model, 85.0% by DEC, and 85.6% by S-DEC model), although S-DIVA model assumes that it might be an estuarine species (probability 100%).

### Phylogeography

A unique COI haplotype of *Novaculina myanmarensis* sp. nov. has been found in the Ayeyarwady River, and four unique COI haplotypes were recorded in the Salween Basin (Table 1). The mean COI *p*-distance (±standard error estimates) between these groups is 0.3 ± 0.1%. Almost all specimens from both rivers share a single 16S rRNA haplotype, with exception of a specimen from the Salween Basin having another haplotype with a single nucleotide substitution (239 G). The 28S rRNA sequences were identical among the samples.

**Table 1:** List of *Novaculina* (Bivalvia: Pharidae) sequences used in this study.

Species	Locality	Voucher no.	COI haplotype code	Acc. numbers of reference sequences			Reference
				*COI*	*16S rRNA*	*28S rRNA*	
*N. gangetica* Benson, 1830	Myanmar: Lemro River	biv_150_1	L1	MF958986	MF958997	MF959011	^4^
		biv_150_2	L2	MF958987	MF958998	MF959012	^4^
		biv_150_3	K1	MF958988	MF958999	MF959013	^4^
	Myanmar: Kaladan River	biv_151_1	K1	MF958989	MF959000	MF959014	^4^
		biv_151_2	K2	MF958990	MF959001	MF959015	^4^
		biv_151_3	K3	MF958991	MF959002	MF959016	^4^
*N. myanmarensis* sp. nov.	Myanmar: Donthami River	biv_369_1	D1	MH670876	MH670886	MH664920	This study
		biv_369_2	D2	MH670877	MH670887	MH664921	This study
		biv_369_3	D3	MH670878	MH670888	MH664922	This study
		biv_369_4	D4	MH670879	MH670889	MH664923	This study
		biv_369_5	D3	MH670880	MH670890	MH664924	This study
	Myanmar: Ayeyarwady River	biv_420_1	A1	MH670881	MH670891	MH664925	This study
		biv_420_3	A1	MH670882	MH670892	MH664926	This study
		biv_420_4	A1	MH670883	MH670893	MH664927	This study
		biv_420_5	A1	MH670884	MH670894	MH664928	This study
		biv_420_6	A1	MH670885	MH670895	MH664929	This study

## Taxonomy

Family Pharidae H. Adams & A. Adams, 1856

Subfamily Pharellinae Stoliczka, 1870

Type genus: *Pharella* Gray, 1854 

= Novaculininae Ghosh, 1920 syn. nov.

Type genus: *Novaculina* Benson, 1830

Genus *Novaculina* Benson, 1830

Type species: *Novaculina gangetica* Benson, 1830 (by monotypy).

### *Novaculina myanmarensis* sp. nov

**Figure 3:**
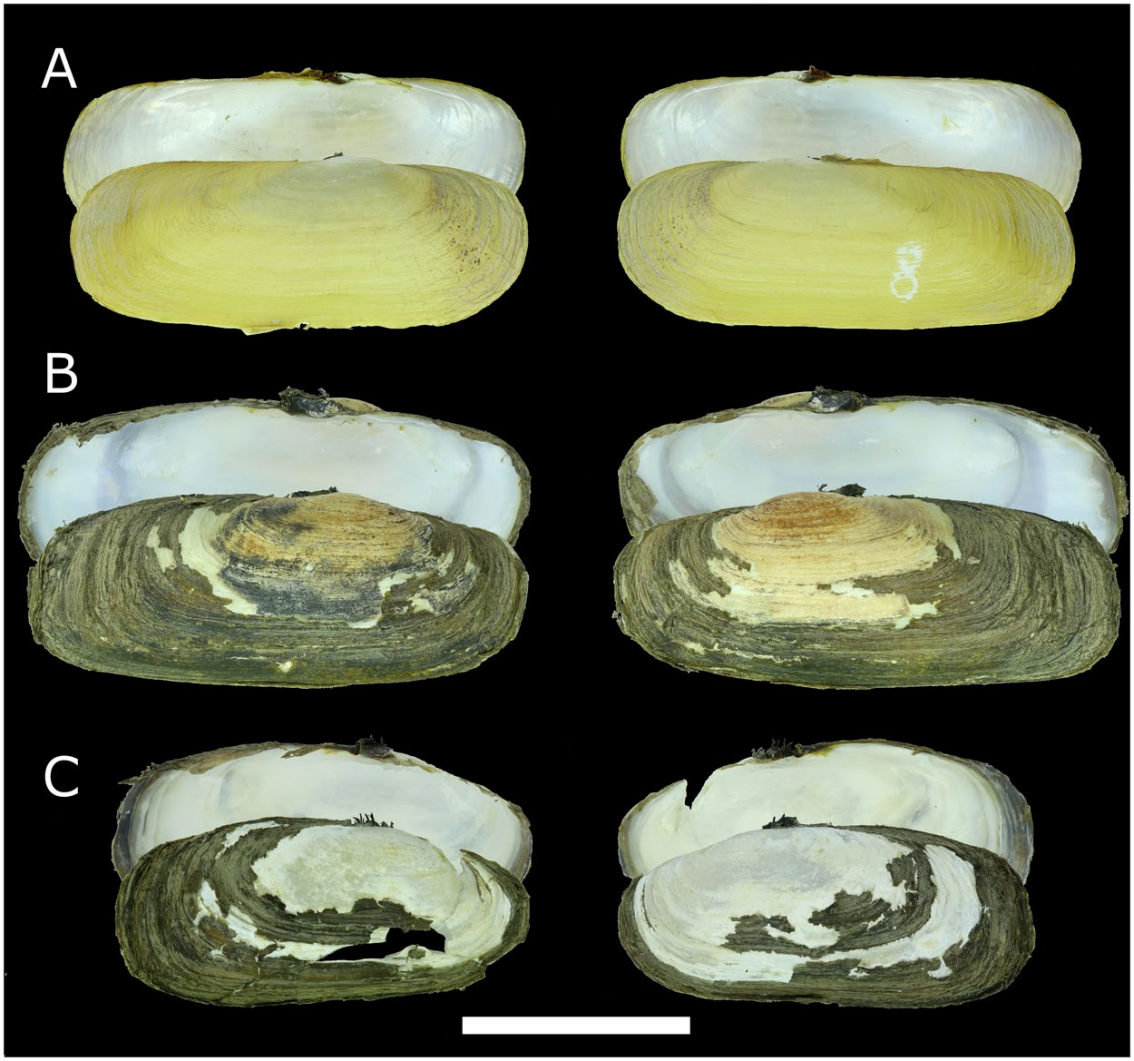
Shells of *Novaculina* spp. (**A**) *N. myanmarensis* sp. nov., holotype RMBH no. biv0420_8, Ayeyarwady River near Thin Baw Kone village, Pakokku Region, Myanmar. (**B**) *N. myanmarensis* sp. nov., paratype RMBH no. biv0369_3, Donthami River, Salween River basin, Myanmar. (**C**) *N. gangetica*, RMBH no. biv0150_24, Lemro River, Myanmar. Scale bar = 2 cm. (Photos: Ekaterina S. Konopleva).

**Figure 4:**
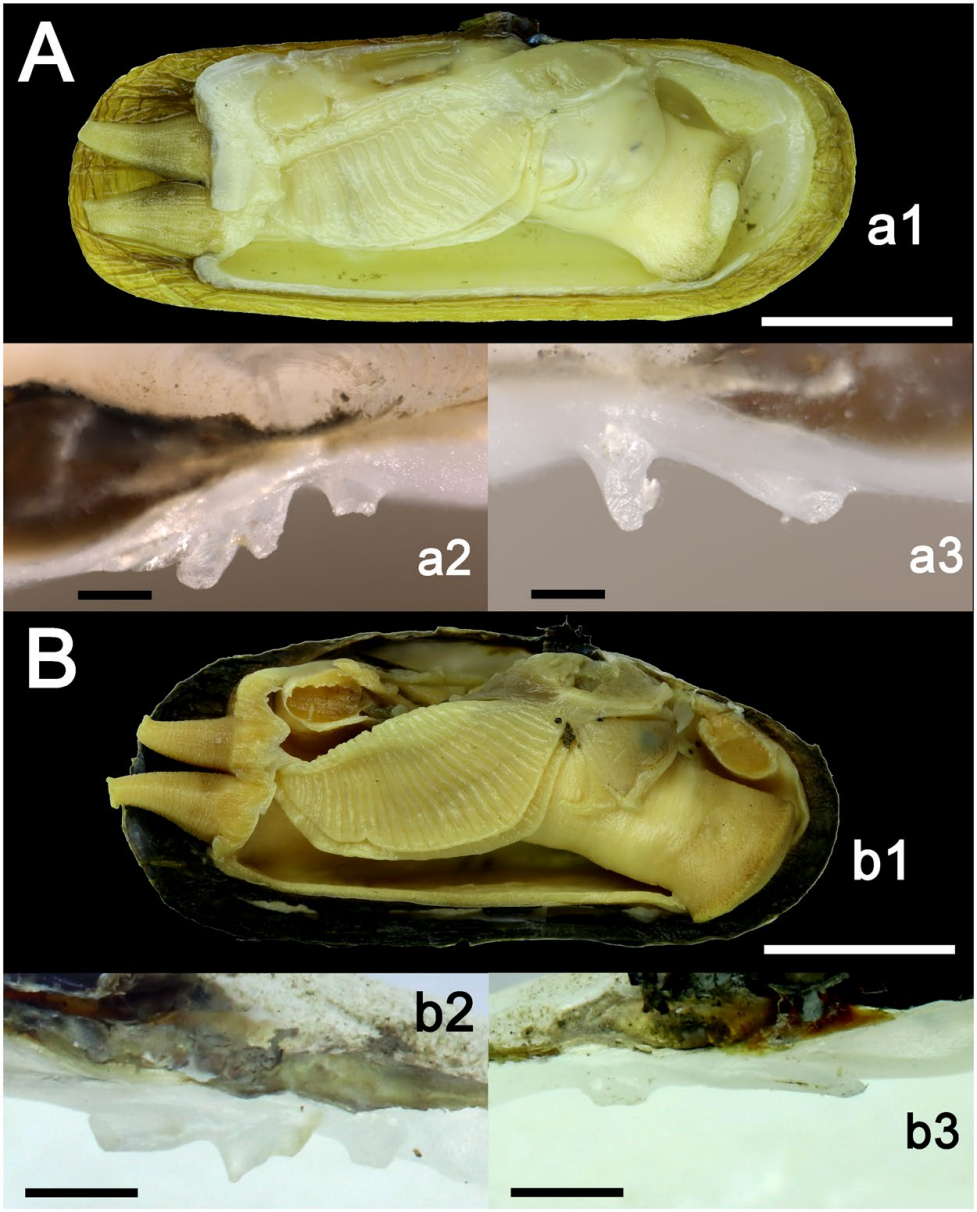
Soft body morphology (right valve and corresponding mantle tissue were removed) and hinge structure of *Novaculina* spp. (**A**) *N. myanmarensis* sp. nov. (holotype RMBH biv0420_8), including (*a1*) soft body (scale bar = 10 mm), (*a2*) pseudocardinal teeth on the left valve, and (*a3*) pseudocardinal teeth on the right valve (scale bars = 0.4 mm). (**B**) *N. gangetica* (RMBH biv0150_24), including (*b1*) soft body (scale bar = 10 mm), (*b2*) pseudocardinal teeth on the left valve, and (*b3*) pseudocardinal teeth on the right valve (scale bars = 1 mm). (Photos: Ekaterina S. Konopleva and Ilya V. Vikhrev).

**Figure 5:**
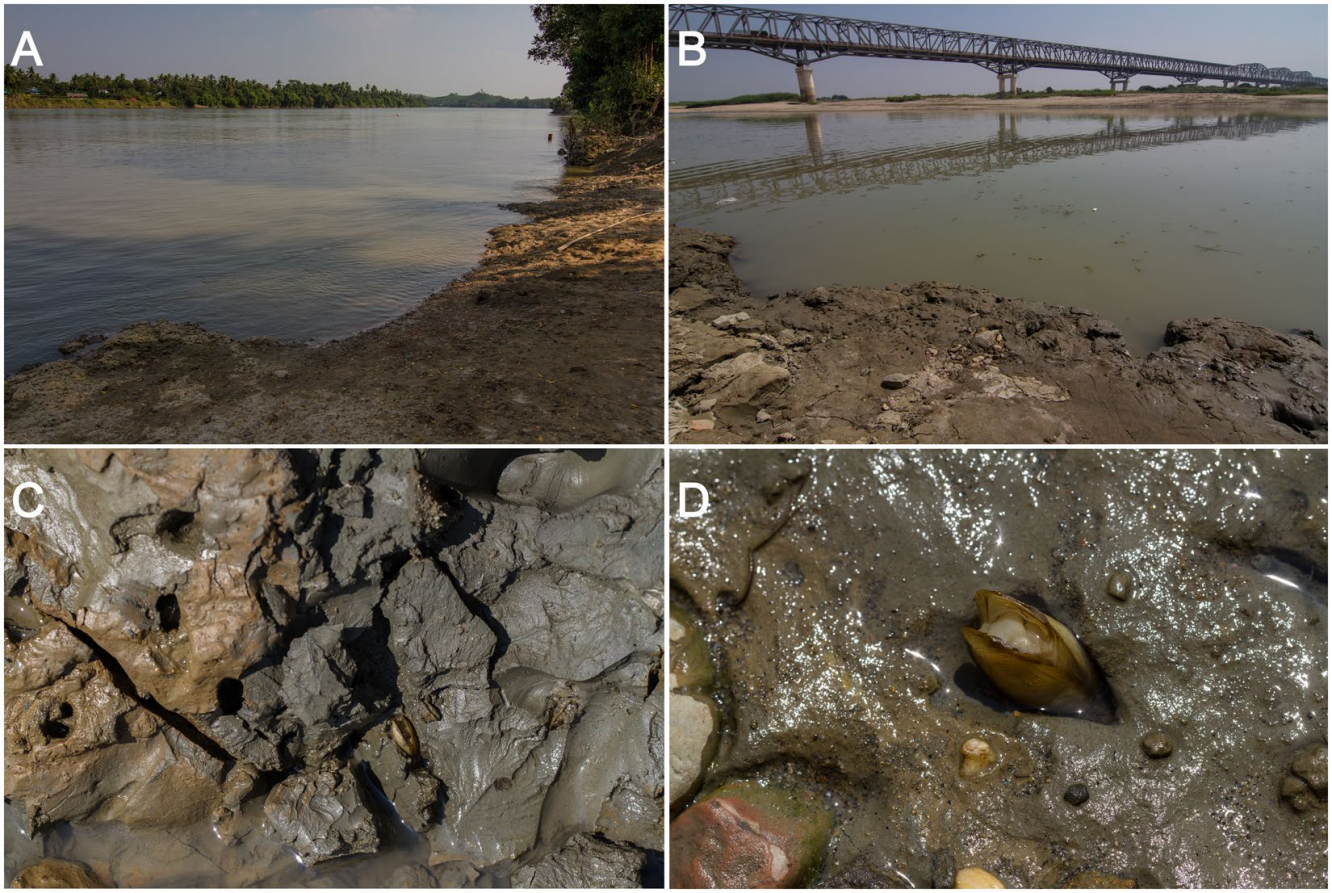
Type locality and habitats of *Novaculina myanmarensis* sp. nov. (**A**) Habitat in the downstream of the Donthami River, 16.6935°N, 97.5819°E. (**B**) Type locality: the middle section of the Ayeyarwady River near Thin Baw Kone village (Pakokku Region), 21.3146°N, 95.0591°E. (**C**) Сlay bottom substrate with clam burrows, the type locality. (**D**) A clam in its burrow, the type locality. (Photos: Ilya V. Vikhrev).

**Figure 6:**
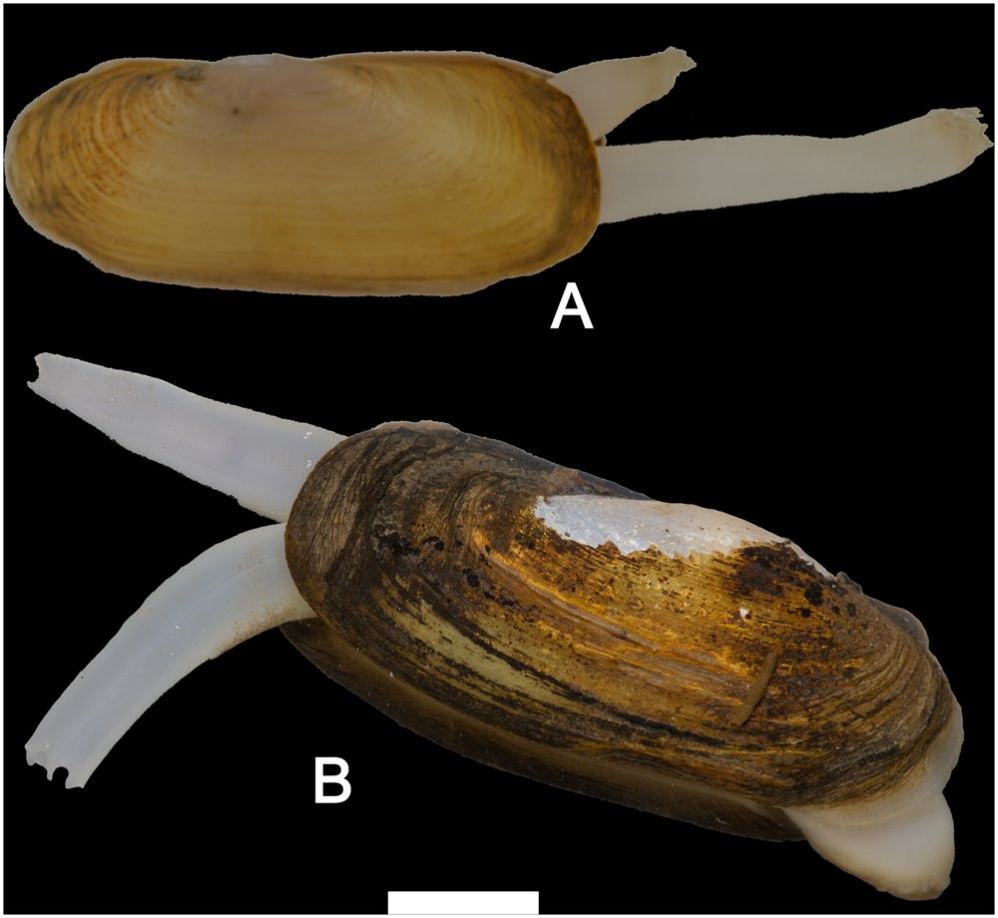
Live *Novaculina* clams with protruded siphons. (**A**) *N. myanmarensis* sp. nov., Donthami River. (**B**) *N. gangetica*, Lemro River. Scale bar = 10 mm. (Photos: Ilya V. Vikhrev).

Figures 3A,B, 4A, 5 and 6A, Tables 1 and 2.

#### Type locality

Myanmar: Ayeyarwady River, near Thin Baw Kone village (Pakokku Region) [21.3146°N, 95.0591°E].

#### Holotype RMBH Biv420_8

Myanmar: Ayeyarwady River, near Thin Baw Kone village (Pakokku Region), clay bottom near the river shore, 21.3146°N, 95.0591°E, 2 March 2018, Bolotov, Vikhrev, Zau Lunn, Nyein Chan, and locals leg.

#### Paratypes

Myanmar: Type locality, same label data, 47 specimens [RMBH Biv0420]; downstream of Donthami River, hard gravel-clay bottom, 16.6935°N, 97.5819°E, 11 February 2018, 5 specimens, local collector leg. [RMBH Biv0369]; Magway Division, Ayeyarwady River, large sandbar 1/2 mi SE of Nyaung-U, 21.2066°N, 94.9062°E, November 2009, 3 specimens, C. N. Piotrowski leg. [CAS 180843]; Ayeyarwady River, near Minbu, 20.1911°N, 94.8788°E, 29 April 2018, 4 specimens, Nyein Chan leg. [FFI].

#### Etymology

The name of this species is derived from the country of Myanmar.

#### Conchological diagnosis

Shell length 20.5–46.5 mm, shell height 7.9–17.5 mm, shell width 4.5–13.3 mm (*N* = 54, Table 2). This species has an elongated shell, and is closely related to *N. gangetica* and *N. chinensis*, but it can be distinguished from these taxa by a more rectangular shell shape with truncated posterior end (vs more oval shell shape with rounded posterior end).

**Table 2:** Measurements of the type series of *Novaculina myanmarensis* sp. nov.

Locality	Status of specimen	Voucher no.*	Shell length, mm	Shell height, mm	Shell width, mm
Myanmar: Ayeyarwady River, Pakokku Region, near Thin Baw Kone village	Holotype	Biv0420_8	40.6	15.2	9.8
	Paratype	Biv0420_1	34.5	12.8	7.9
	Paratype	Biv0420_2	38.1	13.9	8.7
	Paratype	Biv0420_3	36.4	12.9	8.7
	Paratype	Biv0420_4	34.9	11.8	7.8
	Paratype	Biv0420_5	34.1	12.3	7.7
	Paratype	Biv0420_6	38.5	13.9	8.5
	Paratype	Biv0420_7	33.7	12.2	7.4
	Paratype	Biv0420_9	40.6	15.2	9.8
	Paratype	Biv0420_10	30.3	11.1	6.4
	Paratype	Biv0420_11	31.4	11.9	7.0
	Paratype	Biv0420_12	35.2	12.4	8.1
	Paratype	Biv0420_13	27.6	10.0	5.8
	Paratype	Biv0420_14	35.4	12.9	7.9
	Paratype	Biv0420_15	33.5	12.2	7.3
	Paratype	Biv0420_16	27.9	10.9	6.1
	Paratype	Biv0420_17	30.3	11.0	6.7
	Paratype	Biv0420_18	36.3	12.5	8.0
	Paratype	Biv0420_19	29.6	11.0	7.0
	Paratype	Biv0420_20	36.4	12.2	7.5
	Paratype	Biv0420_21	30.9	11.3	6.5
	Paratype	Biv0420_22	34.1	12.7	8.0
	Paratype	Biv0420_23	33.4	12.0	7.0
	Paratype	Biv0420_24	33.1	11.4	7.1
	Paratype	Biv0420_25	29.8	10.5	6.7
	Paratype	Biv0420_26	30.2	11.0	6.5
	Paratype	Biv0420_27	29.1	10.8	6.8
	Paratype	Biv0420_28	32.3	11.8	7.0
	Paratype	Biv0420_29	25.4	9.0	6.0
	Paratype	Biv0420_30	28.3	10.1	6.3
	Paratype	Biv0420_31	26.4	9.7	6.2
	Paratype	Biv0420_32	27.1	10.5	6.1
	Paratype	Biv0420_33	24.3	9.3	5.4
	Paratype	Biv0420_34	24.1	9.5	4.9
	Paratype	Biv0420_35	26.2	10.2	6.3
	Paratype	Biv0420_36	21.6	8.1	4.9
	Paratype	Biv0420_37	28.5	11.6	5.6
	Paratype	Biv0420_38	20.5	7.9	4.5
	Paratype	Biv0420_39	25.0	9.5	5.8
	Paratype	Biv0420_40	38.1	14.2	9.0
	Paratype	Biv0420_41	40.4	15.0	9.3
	Paratype	Biv0420_42	35.6	12.8	7.9
	Paratype	Biv0420_43	34.3	12.5	7.6
	Paratype	Biv0420_44	33.5	11.4	7.1
	Paratype	Biv0420_45	33.1	11.4	6.9
	Paratype	Biv0420_46	32.3	12.0	7.5
	Paratype	Biv0420_47	36.8	12.7	7.9
	Paratype	Biv0420_48	26.5	10.0	5.6
Myanmar: downstream of Donthami River	Paratype	Biv0369_1	46.5	17.0	12.0
	Paratype	Biv0369_2	41.9	16.2	11.0
	Paratype	Biv0369_3	43.9	17.0	13.3
	Paratype	Biv0369_4	43.3	17.3	12.9
	Paratype	Biv0369_5	42.6	17.5	12.3
		**Mean ± s.e.m**.	**32.91 ± 0.81**	**12.12 ± 0.31**	**7.58 ± 0.27**

*RMBH – Russian Museum of Biodiversity Hotspots, Federal Center for Integrated Arctic Research, Russian Academy of Sciences (Arkhangelsk, Russia).

#### Molecular diagnosis

The new species differs from *N. gangetica* by the fixed nucleotide substitutions: 49 substitutions in the COI gene fragment [29 G, 38 A, 53 G, 59 A, 92 A, 128 C, 134 C, 161 G, 170 T, 173 A, 182 A, 185 A, 197 A, 200 A, 212 G, 213 C, 215 T, 230 A, 243 T, 245 A, 254 A, 299 A, 302 A, 308 T, 311 C, 314 G, 329 G, 347 T, 362 G, 371 G, 377 C, 380 A, 404 G, 413 G, 464 T, 467 G, 470 G, 485 G, 491 G, 497 T, 521 G, 545 A, 551 G, 560 G, 569 A, 572 A, 590 T, 617 C, 644 A], 8 substitutions in the 16S rRNA gene fragment [24 G, 74 G, 237 A, 239 G, 242 A, 287 G, 288 T, 305 A], and one substitution in the nuclear 28S rRNA gene fragment [429 A].

#### Description

Shell shape from rectangular to oval-elongated, dorsal and ventral margin are almost parallel to each other (Fig. 3A,B). Shell thin or moderately thick, not inflated. Periostracum from light yellow to brown; nacre whitish, shining. Umbo more or less prominent, in the first half of the shell. Pseudocardinal teeth small and distant from each other, two on the right valve and three on the left valve. Anterior muscle scar pyriform, posterior muscle scar shallow and with rounded shape. The mantle and its edges colored in light yellow. The gills elongated and ribbed (Fig. 4A). The anterior margin of inner gills slightly longer and wider than the outer gills. Foot stumpy, slightly dilated at the end and somewhat truncated; branchial siphon stouter than the anal one, almost the same length, surface of siphon ribbed (Figs 4A and 6A).

#### Intraspecific conchological variability

Specimens from the Donthami and Ayeyarwady rivers are rather different from each other conchologically (compare Fig. 3A,B). The first ones are stronger and thicker, have more truncated posterior end, slightly concave dorsal margin, more developed umbo and hinge. The specimens from the Ayeyarwady River are characterized by more elliptical and very thin shell with light-yellow and smoother periostracum. At first glance, these conchological differences may reflect an environment-induced variability, because the populations were recorded from sites with different bottom substrate (i.e. soft clay substrate in the Ayeyarwady River vs hard gravel-clay substrate in the Donthami River).

#### Distribution

Donthami (Salween River basin) and Ayeyarwady rivers in Myanmar.

#### Habitat

Downstream and middle section of large rivers, in fresh water (Fig. 5A,B). This species inhabits gravel-clay and clay bottom, in which it makes deep vertical holes (Fig. 5C,D).

#### Comments

Local villagers harvest *N. myanmarensis* sp. nov. from the downstream section of the Donthami River (food for consumption). In contrast, this species seems to be unutilized in the Ayeyarwady River.

### *Novaculina gangetica* Benson, 1830

*Novaculina gangetica* Benson^16^: p. 63; Subba Rao^7^: p. 223; Graf^2^: p. 152.

Figures 3C, 4B, and 6B.

#### Type locality

Ganges, Calcutta [India, approximately 22.6°N, 88.3°E].

#### Type series

The University Museum of Zoology, Cambridge, UK [UMZC I.102125: eleven syntypes from the Robert McAndrew collection, labeled “Bens. Coll., Ganges, Calcutta”].

#### Conchological diagnosis

Shell length 28.1–39.7 mm, shell height 12.9–17.5 mm, shell width 8.1–12.7 mm (*N* = 24). This species has an elongated shell, and is closely related to *N. myanmarensis* sp. nov. and *N. chinensis*. It could be distinguished from *N. myanmarensis* sp. nov. by a more ovate shell shape with rounded posterior end (vs more rectangular shell shape with truncated posterior end). *N. gangetica* differs from *N. chinensis* by somewhat higher and shorter shell with slightly convex ventral margin (vs more elongated shell with straight ventral margin).

#### Intraspecific conchological variability

Some specimens have somewhat trapezoidal shell, with slightly expanded posterior end^4^.

#### Distribution

Ganges River and its tributaries in India and Bangladesh ranging from the delta to at least 1,500 km upstream^7,8,17,18^, Buriganga and Pashur river systems in Bangladesh^19,20^, and Kaladan and Lemro rivers in Myanmar^4^.

#### Habitat

The species inhabits downstream and middle sections of large rivers, in fresh or slightly brackish water^4,8^. *N. gangetica* prefers clay bottom, in which it makes cylindrical holes^4,16,17^, but it was also recorded in soft sand and silt bottom^8^. In the Kaladan River, this species also inhabits submerged rocks, in which it was recorded from the vacant borings of *Lignopholas fluminalis*, filled with clay^4^. Benson^16^ noted that this species rarely occurs from holes in rocks in the Jumna and Gumti rivers, and that the specimens from such a habitat have an asymmetrical shell.

#### Comments

Local villagers harvest *N. gangetica* from the Kaladan and Lemro rivers in Myanmar (food for consumption and local market trade), but it seems to be unutilized in India^7^.

### *Novaculina siamensis* Morlet, 1889

*Novaculina siamensis* Morlet^10^: p. 172, 198; Brandt^9^: p. 303; Graf^2^: p. 152; Sayenko *et al*.^11^: p. 182.

#### Type locality

Marais de Chantakam (Siam)^10^ [M. Chantakam, rainfall station on a tributary of the Phra Prong River^21,22^, Thailand, approximately 14.0°N, 102.0°E].

#### Type series

Whereabouts unknown. Morlet’s collection of shells from Indochina went to P. Dautzenberg and is in the Royal Belgian Institute of Natural History, Brussels, Belgium. However, the type series of *N. siamensis* seems to be lacking in this collection (Thierry Backeljau, pers. comm., 2018).

#### Conchological diagnosis

Shell length 30–38 mm, shell height 13–18 mm, shell width 10–15 mm^9^. This species could be distinguished from all the other *Novaculina* taxa by its much shorter and higher shell, less prominent umbo, clear sculpture with concentric growth lines, and dark yellow periostracum.

Intraspecific conchological variability: Some shells in the Mekong Delta population are asymmetrical and torsed^11^.

#### Distribution

Bang Pakong and Pa Sak River basins in Thailand^9,10^, and the Mekong Delta in Vietnam^11^. We assume that a population of *N*. cf. *siamensis* from a tidal creek in the Trang Province of Thailand^23^ belongs to another species, because this creek empties into the Andaman Sea.

#### Habitat

Upstream section of medium-sized rivers, in fresh water, probably on clay bottom substrate. However, it was found in a slightly brackish, tidal channel in the Mekong Delta^11^.

#### Comments

This species seems to be unutilized in Thailand.

### *Novaculina chinensis* Liu & Zhang, 1979

*Novaculina chinensis* Liu & Zhang^12^: p. 356; Qin^24^: p. 305; He & Zhuang^25^: p. 128; Graf^2^: p. 152; Chen *et al*.^26^: p. 4.

#### Type locality

Wuxi, Jiangsu Province [Lake Taihu, approximately 31.4402°N, 120.3143°E]^12^.

#### Type series

National Zoological Museum of China, Institute of Zoology, Chinese Academy of Sciences, Beijing, China [holotype NZMC KS 747703, paratypes NZMC FM00855]^25^.

#### Conchological diagnosis

Shell length 34–46 mm, shell height 11–16 mm, shell width 8–10 mm^12^. This species is closely related to *N. myanmarensis* sp. nov. and *N. gangetica* by an elongated shell shape, but could be distinguished from these species by more prominent, somewhat acute umbo.

#### Intraspecific conchological variability

Not known.

#### Distribution

Downstream of the Yangtze River, China, most records from Lake Taihu^12,24,27^, Lake Hongze^14^, and Lake Chaohu^13^.

#### Habitat

Large floodplain lakes, in fresh water^12–14,24,27^. A single record from the Shangqing River^15^.

#### Comments

This species seems to be unutilized in China. A parasitic mite species, *Unionicola imamurai* Hevers, 1978, has been reported from *N. chinensis*^15^.

## Discussion

### Taxonomic conclusions

Our results reveal that the genus *Novaculina* comprises four species: *N. gangetica* from India, Bangladesh and northwestern Myanmar, *N. myanmarensis* sp. nov. from central and eastern Myanmar, *N. siamensis* from Thailand and southern Vietnam, and *N. chinensis* from southeastern China (Fig. 1). An additional species, *Novaculina andamanensis*, was described from the Andaman Islands but without a precise locality^28^ (holotype no. ZSI M4060/1, paratype no. ZSI 20765/4 [Subba-Rao^7^ considered the latter specimen to be the holotype], malacological collection of the Zoological Survey of India, Kolkata, India^29^). However, this species has been considered a junior synonym of the marine bivalve species *Azorinus coarctatus*^29,30^. We agree with that taxonomic conclusion, because, according to the original description and figure of the type specimen^28^, this species has somewhat trapezoidal shell with concave ventral margin as seen in *Azorinus coarctatus*.

Records of *Novaculina* are still lacking from the downstream sections of several large and medium-sized Southeast and East Asian rivers such as the Pearl River in China and Red River in Vietnam. Taking into account a poor knowledge of freshwater fauna in these basins, further records of new *Novaculina* taxa could not be ruled out. An occasional record of *Novaculina* cf. *siamensis* from a small creek in southern Thailand^23^ suggests that the members of this genus could also establish permanent populations in small-sized freshwater basins, the fauna of which is almost unknown.

Two pharid genera, i.e. *Sinonovacula* and *Novaculina*, were hitherto placed in the Novaculininae^5^. This subfamily was established for *Novaculina gangetica*^31^, but, later, *Sinonovacula constricta* had also been placed within it^32^. However, we found that *Pharella javanica* belongs to a well-supported clade together with *Sinonovacula* and *Novaculina* species (Fig. 2), as it was also shown by another study^4^. According to this, we propose the Novaculininae as a junior synonym of the Pharellinae. A close similarity between *Pharella* and *Sinonovacula* has also been recorded by a functional morphology, particularly in the presence of crescentric anterior and posterior pedal protractor muscles in both taxa^5^.

### Biogeographic implications

Discovery of the new *Novaculina* species from the Salween and Ayeyarwady river drainage basins in Myanmar indicates that the range of this genus is rather continuous and extends along the continental margin of Asia from the Ganges River to the Yangtze River (Fig. 1). Unfortunately, the phylogenetic affinities of the two eastern species, i.e. *Novaculina siamensis* and *N. chinensis*, are still unknown because of the lack of available molecular data. However, they may represent a divergent clade, because the Thai–Malay Peninsula is a significant biogeographic barrier to longitudinal dispersal of aquatic animals^33^. This barrier could have existed during most of the Cenozoic Epoch^34^, although it may have occasionally been incised, but not breached, at the Isthmus of Kra^33^. Our statistical biogeographic modeling strongly supports the hypothesis^4,6,18^ that the genus *Novaculina* is a relict, marine-derived freshwater clade. Similar examples of such secondary freshwater lineages are known among a variety of other taxa, e.g. in fishes, gastropods, polychaetes, and crustaceans^4,35^.

The high level of molecular divergence between the two western species, i.e. *Novaculina gangetica* and *N. myanmarensis* sp. nov., supports a new freshwater biogeographic division of Southeast Asia that has been developed on the basis of unionid mussel research^36–38^. According to this model, the drainages of the Arakan coast of Myanmar, the Ayeyarwady, Bago, Sittaung, and Bilin river basins, and east to the Salween River and drainages of southern Myanmar belong to the Western Indochina Subregion of the Oriental Region^38^. This subregion has high levels of faunal endemism and is separated well from the Indian and Sundaland subregions^38,39^. However, our new study reveals that the northern drainages of the Aracan coast such as the Kaladan and Lemro rivers seem to be a rather marginal part of the Indian Subregion that has already been shown by another research^4^. Anyway, the presence of sister but highly divergent species in the Ganges and Ayeyarwady rivers even in salt-tolerant freshwater taxa such as *Novaculina* strongly indicates that these basins were separated at least since the Miocene (Fig. 2).

In contrast, a shallow genetic divergence between populations of *Novaculina myanmarensis* sp. nov. from the Salween and Ayeyarwady river basins in Myanmar suggests that there were relatively recent (i.e. Late Pleistocene) dispersal events in this species among the downstream sections of these large river drainages. The phylogeography of freshwater mussels (Unionidae) partly reflects this pattern, e.g. the distribution range of *Leoparreysia tavoyensis* crosses numerous freshwater drainages from the Tavoy (north of the Thai–Malay Peninsula) to the Ayeyarwady^36,37^. However, the majority of unionid species in Myanmar appear to be restricted to certain drainage basins or their tributaries^36,37^.

There are several widespread salt-tolerant estuarine and freshwater species, e.g. a polychaete, *Neanthes meggitti* (Nereididae), and a pholadid bivalve, *Lignopholas fluminalis* (Pholadidae)^4^, the range of which encompasses the downstream sections of the Ganges and Ayeyarwady rivers. Such taxa were described from the delta of Ayeyarwady and later have been discovered from the Ganges, or vice versa, and they are ecologically associated with the typical *Novaculina* habitats^4^. The discovery of a new *Novaculina* species in Myanmar indicates that such taxa with broad distribution may actually represent cryptic species complexes, although this preliminary assumption is in need of future molecular research with extensive field surveys in South and Southeast Asia.

## Methods

### Data sampling and mapping

The samples of *Novaculina myanmarensis* sp. nov. were collected from two localities during a field trip to Myanmar in 2018. Additional materials were investigated in the collections of the Fauna & Flora International – Myanmar Program [FFI] (Yangon, Myanmar) and California Academy of Sciences [CAS] (San Francisco, USA). We processed new COI, 16S rRNA and 28S rRNA sequences from ten specimens of *Novaculina myanmarensis* sp. nov. (Table 1) using the standard approach as described in our previous work^4^. Sequences of *Novaculina gangetica* and other Pharidae taxa were obtained from GenBank (Table 1 and Supplementary Table 1). We collected a dataset of reliable georeferenced records of *Novaculina* species from published sources and museum specimens (Supplementary Table 2). The map was created using ESRI ArcGIS 10 software (www.esri.com/arcgis).

### Morphological study

The samples were studied using a stereomicroscope (Leica M165C, Leica Microsystems, Germany). The comparative analysis of taxa was performed according to the standard conchological patterns, i.e. the shape of shell, hinge structure, muscle attachment scars, and position of umbo.

### Sequence alignment, saturation analyses and congruence of phylogenetic signals

We aligned each gene data set using the MUSCLE algorithm in MEGA6^40^. We performed the saturation test of Xia *et al*.^41^ with DAMBE v. 5.3.108^42^, but we found no evidence of substitution saturation (*P* < 0.001). A partition homogeneity test was calculated in PAUP* v. 4.0a151 to confirm the congruence of phylogenetic signals among sequence data sets^43^. This test revealed that the signals among the data sets are consistent (*P* > 0.1 in all the combinations).

### Phylogenetic analyses

We computed maximum likelihood and Bayesian inference phylogenetic models with RAxML v. 8.2.6 HPC Black Box^44^ and MrBayes v. 3.2.6^45^, respectively. The settings of analyses were as described in Bolotov *et al*.^4^. The best substitution models that were used for the Bayesian analyses are listed in Supplementary Table 3. The phylogenetic analysis was done at the San Diego Supercomputer Center through the CIPRES Science Gateway^46^.

### Divergence time modeling

A time-calibrated phylogenetic model has been calculated with BEAST 1.8.4^47^ using the same substitution models as for the MrBayes analyses (Supplementary Table 3). A lognormal relaxed clock and Yule speciation process with continuous quantile parametrization were assigned as model’s priors. To timing the phylogeny, we used the following new crown fossil calibration: †*Leptosolen otterensis* Cragin (1894)^48^. Diagnosis and phylogenetic placement: Shell thin, elongated, moderately convex, inequilateral, compressed anteriorly, with anterior fold and angular growth lines around anterior and posterior margins. This species seems to be the oldest member of the genus^49^, and may represent an ancestral lineage of the Pharidae. Stratigraphic horizon and locality: dark-gray shale of Kiowa Formation (Albian) in central Kansas^48^. Absolute age estimate: Lower Cretaceous, upper Albian boundary, 100.5 Ma, based on stratigraphy^48^; 95% soft upper bound 113.0 Ma (lower Albian boundary). Prior setting: exponential distribution, mean (lambda) = 3.4, MRCA: *Novaculina gangetica* – *Siliqua radiata*. Five independent runs of 30,000,000 generations were processed, with sampling every 1,000 generation. The resulting tree sets were combined using LogCombiner 1.8.4^47^. An appropriate burn-in was chosen for each tree set with Tracer v. 1.6^50^. A maximum clade credibility tree has been computed with TreeAnnotator 1.8.4 with an additional resampling every 10,000 generation^47^.

### Ancestral area modeling

Ancestral area reconstruction has been performed on the basis of three algorithms, i.e., Statistical Dispersal-Vicariance Analysis (S-DIVA), Dispersal-Extinction Cladogenesis (DEC), and Statistical Dispersal-Extinction Cladogenesis (S-DEC) implemented in RASP v. 3.2^51^ as described in Bolotov *et al*.^4^. We assigned two possible ancestral areas of the in-group species, i.e., (a) estuarine and (ab) freshwater to estuarine. The three primary models were combined into an integrative model using the Combine Results option of RASP v. 3.2^51^.

### Molecular diagnoses

To test the molecular differences between *N. myanmarensis* sp. nov. and *N. gangetica*, we used an approach of Bolotov *et al*.^37^. The mean *p*-distances and number of fixed nucleotide substitutions were accessed using MEGA6^40^.

### Nomenclatural acts

The electronic edition of this article conforms to the requirements of the amended International Code of Zoological Nomenclature (ICZN), and hence the new names contained herein are available under that Code from the electronic edition of this article. This published work and the nomenclatural acts it contains have been registered in ZooBank (http://zoobank.org), the online registration system for the ICZN. The LSID for this publication is: urn:lsid:zoobank.org:pub:19E34605-30C2-4DAB-B81F-53A1FDB324DB. The electronic edition of this paper was published in a journal with an ISSN, and has been archived and is available from PubMed Central.

## Electronic supplementary material

Supplementary Information

## Data Availability

The sequences used in this study are available from GenBank. Accession numbers for each specimen are presented in Table 1. The type series of the new species is available in the Russian Museum of Biodiversity Hotspots [RMBH], Federal Center for Integrated Arctic Research, Russian Academy of Sciences (Arkhangelsk, Russia), Fauna & Flora International – Myanmar Program [FFI] (Yangon, Myanmar), and California Academy of Sciences [CAS] (San Francisco, USA).
