# Supplementary material for: Discovery of Novaculina myanmarensis sp. nov. (Bivalvia: Pharidae: Pharellinae) closes the freshwater razor clams range disjunction in Southeast Asia
Source: Sci Rep. 2018 Nov 5;8:16325. doi: 10.1038/s41598-018-34491-8 (PMC6218526; doi:10.1038/s41598-018-34491-8)
Supplement: Supplementary file 1 — Supplementary Information [file 41598_2018_34491_MOESM1_ESM.pdf]

SUPPLEMENTARY INFORMATION

# Discovery of *Novaculina myanmarensis* **sp. nov.** (Bivalvia: Pharidae: Pharellinae) closes the freshwater razor clams range disjunction in Southeast Asia

Ivan N. Bolotov*, Ilya V. Vikhrev, Manuel Lopes-Lima, Zau Lunn, Nyein Chan, Than Win, Olga V. Aksenova, Mikhail Yu. Gofarov, Alexander V. Kondakov, Ekaterina S. Konopleva & Sakboworn Tumpeesuwan

*Corresponding author: inepras@yandex.ru

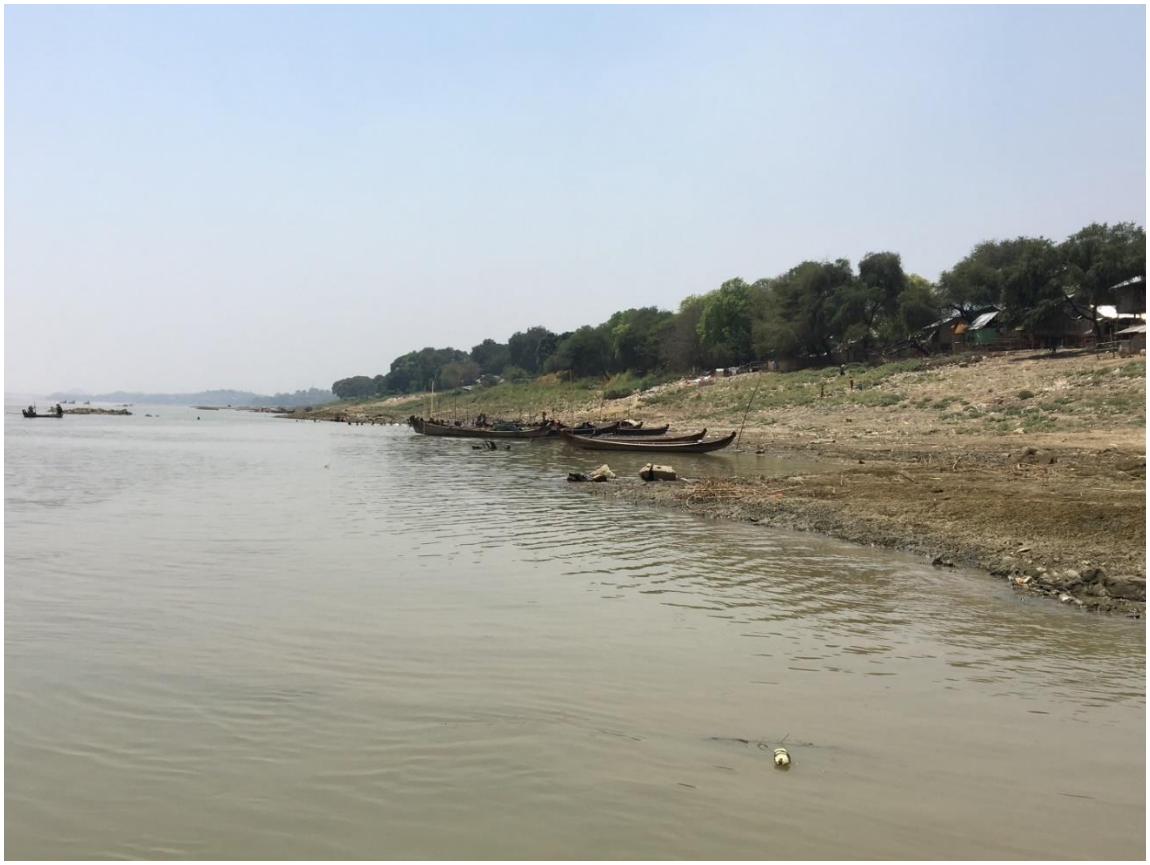

Habitat of *Novaculina myanmarensis* **sp. nov.**, Ayeyarwady River, near Minbu, 20.1911° N, 94.8788° E (Photo: Nyein Chan).

# Contents

**Supplementary Table 1.** List of additional sequences used in this study for phylogenetic reconstructions.

**Supplementary Table 2.** List of available georeferenced records of *Novaculina* species.

**Supplementary Table 3.** Models of sequence evolution for each partition based on corrected Akaike Information Criterion (AICc) of MEGA6 that were applied within Bayesian models.

**Supplementary Table 1.** List of additional sequences from GenBank used in this study for phylogenetic reconstructions. Species names and environmental preference are on the basis of the World Register of Marine Species database[1].

| Taxa | Environment | Locality | *COI* | *16S rRNA* | *28S rRNA* |
|---|---|---|---|---|---|
| *Sinonovacula constricta* (Lam., 1818) | Marine | China | JN859986 | JN859901 | AF131005 |
| *S. mollis* (G. B. Sowerby II, 1874) (=*S. rivularis* Huang & Zhang, 2007) | Marine | China | JN859994 | JN859908 | n/a |
| *Pharella javanica* (Lam., 1818) | Marine | Thailand | n/a | n/a | AM779683 |
| *Cultellus attenuatus* Dunker, 1862 | Marine | China | JN859998 | JN859913 | n/a |
| *Ensiculus cultellus* (L., 1758) | Marine | Queensland, Australia | n/a | n/a | AM779682 |
| *Pharus legumen* (L., 1758) | Marine | South Wales, UK | n/a | n/a | AM779684 |
| *Phaxas pellucidus* (Pennant, 1777) | Marine | Sweden, Tjärnö | KC429145 | KC429309 | KC429508 |
| *Ensis magnus* Schumacher, 1817* | Marine | Ireland & Spain** | EU523690 | AJ586445 | n/a |
| *E. leei* Huber, 2015* | Marine | Europe & USA** | EU523673 | HF970450 | JF909603 |
| *E. macha* (Molina, 1782)* | Marine | Chile** | JF301780 | HF970471 | n/a |
| *Siliqua alta* (Broderip & Sowerby, 1829)* | Marine | Hokkaido, Japan | n/a | AB751362 | AB746908 |
| *S. radiata* (Linnaeus, 1758)* | Marine | China | JN860004 | JN859917 | n/a |

*Outgroup taxa. **Chimeric sequence. n/a – not available.

**Supplementary Table 2.** List of available georeferenced records of *Novaculina* species

| Species | Locality | Latitude | Longitude | Reference |
|---|---|---|---|---|
| *N. myanmarensis* **sp. nov.** | Myanmar: downstream of Donthami River | 16.6935 | 97.5819 | This study (RMBH) |
| *N. myanmarensis* **sp. nov.** | Myanmar: Ayeyarwady River, Pakokku Region, near Thin Baw Kone village* | 21.3146 | 95.0591 | This study (RMBH) |
| *N. myanmarensis* **sp. nov.** | Myanmar: Ayeyarwady River, large sandbar 0.5 mi SE of Nyaung-U | 21.2066 | 94.9062 | This study (CAS) |
| *N. myanmarensis* **sp. nov.** | Myanmar: Ayeyarwady River, near Minbu | 20.1911 | 94.8788 | This study (FFI) |
| *N. gangetica* | India: Ganges River at Vindhyachal upstream of Mirzapur | 25.1666 | 82.5072 | Ref. 5 |
| *N. gangetica* | India: Hooghly River downstream of Howra | 22.4278 | 88.1368 | Ref. 5 |
| *N. gangetica* | India: Ganges River at Varanasi | 25.3279 | 83.0464 | Ref. 6 |
| *N. gangetica* | India: Ganges River at Patna | 25.6589 | 85.1573 | Ref. 7 |
| *N. gangetica* | India: Calcutta* | 22.6000 | 88.3000 | MCZ 224805; UMZC I.102125 |
| *N. gangetica* | India: Jumna [Yamuna] River at Hamirpur | 25.9617 | 80.1525 | Ref. 8 |
| *N. gangetica* | India: Gomti River at Jaunpur | 25.7474 | 82.6874 | Ref. 8 |
| *N. gangetica* | India: Burhi Gandak River at Khagaria | 25.5009 | 86.4776 | Ref. 9 |
| *N. gangetica* | Bangladesh: Turag River at Tangi Bridge | 23.8997 | 90.4081 | Ref. 10 |
| *N. gangetica* | Bangladesh: Moyur River | 22.7719 | 89.5402 | Ref. 11 |
| *N. gangetica* | Myanmar: Kaladan River | 21.0094 | 92.9813 | Ref. 12 |
| *N. gangetica* | Myanmar: Lemro River | 20.6150 | 93.2481 | Ref. 12 |
| *N. siamensis* | Thailand: Chantakam [Bang Pakong River basin]* | 14.0000 | 102.0000 | Ref. 2 |
| *N. siamensis* | Thailand: Pa Sak River near Saraburi | 14.5304 | 100.9113 | Ref. 3 |
| *N. siamensis* | Vietnam: Ba Lai River, Mekong Delta | 10.2647 | 106.4397 | Ref. 4 |
| *N. chinensis* | China: Lake Taihu, Wuxi* | 31.4402 | 120.3143 | Ref. 13, 14 |
| *N. chinensis* | China: Lake Taihu, southwest | 31.1843 | 120.4267 | Ref. 15 |
| *N. chinensis* | China: Lake Hongze | 33.3075 | 118.7100 | Ref. 16 |
| *N. chinensis* | China: Lake Chaohu | 31.5224 | 117.5616 | Ref. 17 |
| *N. chinensis* | China: Yintan | 28.0333 | 117.0667 | Ref. 18 |

*Type locality (see Taxonomic Account for details).

**Supplementary Table 3.** Models of sequence evolution for each partition based on corrected Akaike Information Criterion (AICc) of MEGA6[19] that were applied within Bayesian models

| Data set and partition | Model | Gamma | Invariant |
|---|---|---|---|
| 1st codon of *COI* | TN93+G | 0.10 | n/a |
| 2nd codon of *COI* | HKY+G | 0.05 | n/a |
| 3rd codon of *COI* | HKY+G | 0.87 | n/a |
| *16S rRNA* | GTR+G+I | 0.40 | 0.51 |
| *28S rRNA* | GTR+G+I | 7.15 | 0.58 |

n/a – not available.

## Supplementary references


1. *World Register of Marine Species* http://marinespecies.org (2018).
2. Morlet, L. Catalogue des Coquilles recueillies, par M. Pavie, dans le Cambodge et le Royaume de Siam, et description d'espèce nouvelles. *Journal de Conchyliologie* **37**, 121–199 (1889).
3. Brandt, R. A. M. The non-marine aquatic mollusca of Thailand. *Archiv für Mollusckenkunde* **105**, 1–423 (1974).
4. Sayenko, E.M., Quang, N.X. & Lutaenko, K.A. Bivalves of the Ba Lai River – one of estuary of the Mekong Delta, Vietnam. in *Life-Supporting Asia-Pacific Marine Ecosystems, Biodiversity and Their Functioning* (ed. Dautova, T.N, Sun, X., Sun, S. & Adrianov, A.V.) 178–184 (Science Press Beijing, 2017).
5. Nesemann, H. A., Sharma, S. U., Sharma, G. O. & Sinha, R. K. Illustrated checklist of large freshwater bivalves of the Ganga river system (Mollusca: Bivalvia: Solecurtidae, Unionidae, Amblemidae). *Nachrichchtenblatt der Ersten Vorarlberger Malakologischen Gesellschaft* **13**, 1–51 (2005).
6. Subba Rao, N.V. *Handbook of freshwater molluscs of India* (Calcutta, 1989).
7. Nesemann, H., Sharma, G. & Sinha, R. Benthic macro-invertebrate fauna and "marine elements" sensu Annandale (1922) highlight the valuable dolphin habitat of river Ganga in Bihar-India. *Taprobanica* **3,** 18–30; DOI:10.4038/tapro.v3i1.3230 (2011).
8. Benson, W.H. Characters of *Tanysiphon*, a new genus of fluviatile shells, allied to Myacidae. *Annals and Magazine of Natural History (3rd series)* **1**, 407–410 (1858).
9. Prabhakar, A. K. & Roy, S. P. Taxonomic diversity of shell fishes of Kosi Region of North-Bihar (India). *The Ecoscan* **2**, 149–156 (2008).
10. Baki, M. A., Hossain, M. M. & Bhouiyan, N. A. Checklist of freshwater mollusca (Gastropoda and Bivalvia) recorded from the Buriganga and Turag rivers, Dhaka, Bangladesh. *The Festivus* **48,** 221–228 (2016).

11. Khan, A. N., Kamal, D., Mahmud, M. M., Rahman, M. A. & Hossain, M. A. Diversity, distribution and abundance of benthos in Mouri River, Khulna, Bangladesh. *International Journal of Sustainable Crop Production* **2**, 19–23 (2007).

12. Bolotov, I. N. et al. Discovery of a silicate rock-boring organism and macrobioerosion in fresh water. *Nature Communications* **9**, 2882; DOI:10.1038/s41467-018-05133-4 (2018).

13. Liu, Y. Y. & Zhang, W. Z. A new species of freshwater razor clam, *Novaculina chinensis*, from Jiangsu Province, China. *Acta Zootaxonomica Sinica* **4**, 356–357 (1979).

14. Qin, B. (Ed.). Lake Taihu, China: Dynamics and environmental change. *Monographiae Biologicae* **87**, 1–348 (2008).

15. Ji, L., Song, C., Cao, X., Zhou, Y. & Deng, D. Spatial variation in nutrient excretion by macrozoobenthos in a Chinese large shallow lake (Lake Taihu). *Journal of Freshwater Ecology* **30**, 169–180; DOI:10.1080/02705060.2014.997816 (2015).

16. Hu, Z., Sun, X., Cai, Y., Guo, L., Chen, Q., Liu, T., Shi, F. & Yang, L. The habitat type and trophic state determine benthic macroinvertebrate communities in lowland shallow lakes of China. *Journal of Limnology* **75**, 330–339; DOI:10.4081/jlimnol.2016.1220 (2016).

17. Cai, Y., Gong, Z. & Xie, P. Community structure and spatiotemporal patterns of macrozoobenthos in Lake Chaohu (China). *Aquatic Biology* **17**, 35–46; DOI:10.3354/ab00455 (2012).

18. Wen, C. & Zhu, Z. Seven species of water mites in the genus *Unionicola* from Jiangxi (Acari: Unionicolidae). *Acta Zootaxonomica Sinica* **24**, 30–37 (1999).

19. Tamura, K., Stecher, G., Peterson, D., Filipski, A. & Kumar, S., MEGA6: Molecular Evolutionary Genetics Analysis version 6.0. *Molecular Biology and Evolution* **30**, 2725–2729; DOI:10.1093/molbev/mst197 (2013).
